# Associations of poor oral health with frailty and physical functioning in the oldest old: results from two studies in England and Japan

**DOI:** 10.1186/s12877-021-02081-5

**Published:** 2021-03-18

**Authors:** Viviana Albani, Kensuke Nishio, Tomoka Ito, Eftychia Kotronia, Paula Moynihan, Louise Robinson, Barbara Hanratty, Andrew Kingston, Yukiko Abe, Michiyo Takayama, Toshimitsu Iinuma, Yasumichi Arai, Sheena E. Ramsay

**Affiliations:** 1 Population Health Sciences Institute, Faculty of Medical Sciences, Newcastle University, Newcastle upon Tyne, UK; 2 Department of Complete Denture Prosthodontics, Nihon University School of Dentistry, Tokyo, Japan; 3 Adelaide Dental School, Faculty of Health and Medical Sciences, The University of Adelaide, Adelaide, Australia; 4 Centre for Supercentenarian Medical Research, Keio University School of Medicine, Tokyo, Japan; 5 Centre for Preventive Medicine, Keio University School of Medicine, Tokyo, Japan

**Keywords:** Frailty, Oldest old, Oral health, Mobility

## Abstract

**Background:**

Very few studies have examined the relationship of oral health with physical functioning and frailty in the oldest old (> 85 years). We examined the association of poor oral health with markers of disability, physical function and frailty in studies of oldest old in England and Japan.

**Methods:**

The Newcastle 85+ Study in England (*n* = 853) and the Tokyo Oldest Old Survey on Total Health (TOOTH; *n* = 542) comprise random samples of people aged > 85 years. Oral health markers included tooth loss, dryness of mouth, difficulty swallowing and difficulty eating due to dental problems. Physical functioning was based on grip strength and gait speed; disability was assessed as mobility limitations. Frailty was ascertained using the Fried frailty phenotype. Cross-sectional analyses were undertaken using logistic regression.

**Results:**

In the Newcastle 85+ Study, dry mouth symptoms, difficulty swallowing, difficulty eating, and tooth loss were associated with increased risks of mobility limitations after adjustment for sex, socioeconomic position, behavioural factors and co-morbidities [odds ratios (95%CIs) were 1.76 (1.26–2.46); 2.52 (1.56–4.08); 2.89 (1.52–5.50); 2.59 (1.44–4.65) respectively]. Similar results were observed for slow gait speed. Difficulty eating was associated with weak grip strength and frailty on full adjustment. In the TOOTH Study, difficulty eating was associated with increased risks of frailty, mobility limitations and slow gait speed; and complete tooth loss was associated with increased risk of frailty.

**Conclusion:**

Different markers of poor oral health are independently associated with worse physical functioning and frailty in the oldest old age groups. Research to understand the underlying pathways is needed.

**Supplementary Information:**

The online version contains supplementary material available at 10.1186/s12877-021-02081-5.

## Background

Rapid rates of population ageing globally represent enormous challenges for the health and social care needs of an increasingly ageing population. In the UK, the number of people 85 years and above is projected to increase by 74% between 2019 and 2039 [1]. Countries such as Japan similarly show sharp increases in the proportion of oldest old [2]. Declines in physical function are critical issues in older populations, with estimated 22–30% of people aged > 80 years being frail [3], and as much as 15% of individuals > 90 years having mobility limitations [4]. Frailty has been defined as a syndrome or systemic condition reflecting an individual’s vulnerability to minor stressors, which increases the risk of adverse outcomes including mortality, disability, falls, hospitalisation and long-term care [3, 5]. Poor physical function (e.g. muscle weakness, limited mobility) are often early manifestations of frailty and in the very old is a risk factor for mortality and morbidity, both independently and through its association with frailty [5].

Poor oral health, also common in older populations, is implicated as an important contributor in the pathway to frailty and limitations in physical function [6, 7]. Dental diseases such as periodontal (gum) disease can be both a cause and manifestation of chronic inflammatory processes which also underlie disability and frailty [8]. Moreover, deterioration of oral health, such as difficulties swallowing, dry mouth (xerostomia) and excessive tooth loss, have profound implications for how well people eat and what they choose to eat [9], potentially affecting diet quality, (e.g low protein intake) [10] and in turn muscle mass, muscle strength and physical performance [11].

To date, the vast majority of the literature on the associations between oral health and physical function in older populations has focused on complete tooth loss or lacking a functional number of teeth (21 or more teeth). Having no- or few teeth has been linked with frailty [8, 12–14], disability in activities of daily living (ADL) [15], mobility limitations [16, 17], and declining gait speed [18]. Amongst other oral health markers, subjective reports of oral health provide strong indications of oral disease in older populations [19], but less research attention has been given to dry mouth and perceived difficulty eating foods due to dental problems. Exceptions include associations of dry-mouth with frailty [14], slow gait speed and mobility limitations [16]; and eating difficulties because of oral health problems with disability [16]. In contrast, after adjustment for health conditions and lifestyle behaviours, no association was found between perceived difficulty eating and functional disability or ADL [15]. Given their relative ease of assessment, more research is needed on whether these oral health indicators could be useful predictive tools for physical function and frailty in older populations. The aim of this cross-sectional study was therefore to examine associations of a range of objective and subjective oral health markers with frailty and physical function measures. We explored these relationships using comprehensive and comparable data of people aged > 85 on physical functioning and oral health in England and Japan: the Newcastle 85+ study, and the Tokyo Oldest Old Survey on Total Health (TOOTH).

## Methods

### Data

The Newcastle 85+ Study is a prospective cohort of people born in 1921 in Newcastle upon Tyne and North Tyneside (North East England) [20]. Participants were recruited in 2006–07 irrespective of their health state (except for terminal illness), living at home or in an institution. The TOOTH study is an observational cohort that randomly recruited people aged ≥85 years resident in Tokyo (Japan) in 2008 [21]. Both studies conducted multidimensional health assessments on a range of biological and social factors associated with health and functioning. Data collection was carried out by trained research nurses (Newcastle 85+), and trained interviewers, dentists and geriatricians (TOOTH) in participants’ homes and/or during clinic visits (TOOTH). The Newcastle 85+ Study collected information on health conditions and medication use from general practice record reviews (GPRR). The Newcastle 85+ Study followed the Code of Ethics of the World Medical Association (Declaration of Helsinki). Newcastle & North Tyneside Local Research Ethics Committee 1 approved the Newcastle 85+ Study. The Keio University School of Medicine (N0.20070047, Dec 2007) and Nihon University School of Dentistry (No.2003–20, 2008) approved the TOOTH study. TOOTH is registered in the University Hospital Medical Information Network Clinical Trial Registry (ID UMIN000001842). Able participants provided informed consent, with signed consultee approval from those participants lacking capacity. Participants from the baseline surveys (Newcastle 85+ Study *n* = 853; TOOTH study *n* = 542) were included in the present analysis.

### Frailty, physical function, and mobility limitation

Participants underwent a physical examination at baseline when recruited into the studies. Frailty status was based on the Fried Frailty Phenotype [22] using data from questionnaires and physical assessments. Participants were coded as “frail” if meeting at least three of the five criteria of the Fried Frailty Phenotype Score (FFS); which included, in the Newcastle 85+ Study, unintentional weight loss, poor energy/endurance (self-reported question “do you feel full of energy”), low physical activity, weakness (grip strength), and slow gait speed (Timed-up-and-go or TUG test) [23]. The same criteria were applied in the TOOTH study, except for poor energy/endurance which was based on the question “I have felt active and vigorous” (WHO-5 Well-being Index) [24] (Table A1 in online supplementary material).

Grip strength (GS) was assessed twice for each hand, alternating between hands, following a standardised protocol [25] using a Takei A5401 digital 0e100 kg × 0.1 kd LCD hand-held dynamometer. The TOOTH study used two measurements of grip strength of the dominant hand using a Tanita 6103 handheld dynamometer (Tanita cooperation, Tokyo, Japan). Walking speed was based on the TUG test using previously described methods to measure time taken to walk three meters [21, 23]. Participants in both studies were classified as having mobility limitations if they reported difficulty getting around in the house, or going up and down stairs/steps, or walking at least 400 yards.

### Oral health markers

The Newcastle 85+ study included a count of number of natural teeth as part of the physical examination of participants conducted by trained research nurses, as well as a question on the age at which the last remaining natural tooth was lost. Other oral health measures assessed through self-report questionnaires included dry mouth symptoms, difficulty swallowing, and difficulty eating foods because of problems with teeth or the mouth. Questions on dryness of mouth included sipping water through the night, taking liquids to help swallow dry foods, feelings of dry mouth when eating, and difficulty swallowing foods because of dry mouth. Report to any one of these questions was taken as presence of dry mouth symptoms. Difficulty swallowing was assessed through questions on difficulty swallowing foods due to dryness of mouth or any other reason, or difficulty swallowing liquids. Ten food items were listed to assess difficulty eating because of problems with teeth and mouth: crusty bread, toast, tomato, raw carrots, roast potato, cooked green vegetables, lettuce, well-done steak, apples and nuts. Responses included “eat easily”, “with some difficulty”, “could not eat at all”.

In the TOOTH study, a count of natural teeth was conducted by a dentist as part of the physical examination. Dryness of mouth was based on measures of salivary volume. Saliva was collected by spiting method for 3 min [26]. Subjective measures of oral health included difficulties swallowing (“How often are you able to swallow comfortably”) [27] and chewing ability. Chewing ability was assessed from the ability to eat 15 foods differing in texture and hardness (easy to eat, eaten with effort, or impossible to eat): boiled rice, udon noodles, tuna sashimi, bananas, and boiled spinach (soft and easy to chew), roasted rice cake, sliced cabbage, hamburger, pickled Chinese cabbage, and rolled boiled fish (slightly difficult to chew), and hard rice crackers, peanuts, yellow pickled radish, boiled bamboo shoots, and pork cutlets (hard and difficult to eat).

### Covariates

Socioeconomic position was based on UK Office for National Statistics Socio-economic Classification (Newcastle 85+ Study) or number of years of education (TOOTH study). Information on smoking and alcohol intake was collected as part of the study interviews and have been previously described [21, 28]. Information on health conditions in the Newcastle 85+ Study from GPRRs included history of cardiovascular disease, diabetes, Alzheimer’s disease, Parkinson’s disease, hypertension, arthritis, osteoporosis, stroke, transient ischaemic attack, respiratory disease, and cancer in the last 5 years. Information on these co-morbidities was collected in the TOOTH study as part of the interview by experienced geriatricians [21]. Cardiovascular disease (CVD) included stroke, transient ischaemic attack (TIA), myocardial infarction, and angina. Hypertension was defined as current use of medication for hypertension, self-reported diagnosis, or systolic blood pressure higher than 140 mmHg at baseline examination. Diabetes was defined as fulfilling one or more criteria: self-reported diagnosis, administration of insulin or other oral hypoglycemic medications, random plasma glucose ≥200 mg/dL, or hemoglobin A1c ≥6.5%. In the Newcastle 85+ Study, GPRR records were used to ascertain regular use of prescribed medications with xerostomia (dry mouth) side effects. These medications were derived and classified using the British National Formulary (BNF https://bnf.nice.org.uk). The medications included antimuscarinics (anticholinergics), antidepressants (selective serotonin reuptake inhibitors, tricyclics), antihistamines, alpha-blockers, antipsychotics, skeletal muscle relaxants (baclofen, tizanidine), centrally acting anti-hypertensives (clonidine), opioids, and diuretics (thiazides and related diuretics, potassium sparing diuretics, etc.). Information on depression was obtained through the Geriatric Depression Scale (GDS-15) in the Newcastle 85+ Study, and through the WHO-5 Well-being Index [24] in the TOOTH study.

### Statistical analyses

Responses to questions on difficulty eating because of dental problems in the Newcastle 85+ Study were coded as: “severe difficulty eating” (≥3 of the 10 foods items as ‘could not eat at all’) and “Any trouble eating *hard foods*” (≥3 hard foods “with some difficulty” or “could not eat at all”). Number of teeth was grouped as having at least 21 teeth [29], number of remaining natural teeth (“none”, “1–20,” and “≥21”), and complete tooth loss (edentulism). In the TOOTH study, number of teeth was categorised as complete tooth loss, < 21 teeth, and a three-category variable (0, 1–20, and ≥ 21 teeth); the three category variable best reflected the distribution of tooth loss in the TOOTH Study. Dry mouth was based on the lowest tertile of salivary volume (0.83 g). Difficulty eating was categorised as “severe difficulty eating” (≥4 foods “impossible to eat”). “Any trouble eating *hard foods*” (≥2 hard foods “eaten with effort” or “impossible to eat”).

Slow walking speed was defined as values in the highest tertile of the TUG distribution (Newcastle 85+ Sudy, men: ≥15 s, women: ≥19 s; TOOTH study, men: ≥14.2 s, women: ≥16 s); and low muscle strength as values in the lowest tertile of the grip strength distribution (Newcastle 85+ Study, men: ≤23 kg, women: ≤13 kg; TOOTH study, men: ≤23 kg, women: 14.3 kg).

The relationship of oral health markers with frailty, mobility limitations, grip strength, and gait speed was assessed using logistic regression. Models for dry mouth were specifically adjusted for medications with xerostomia as a side effect (Newcastle 85+ Study) and depression (both studies).

## Results

Table 1 presents the characteristics of the two study populations. For the 853 participants with available baseline data in the Newcastle 85+ Study, prevalence of frailty was 28% and that of mobility limitations was 57%. Notably, 37% of participants reported having any trouble eating hard foods because of dental problems, 57% of the sample experienced dry mouth symptoms, and 55% of participants had no remaining teeth (edentulism), having on average lost their last natural tooth at the age of 42 years (SD = 17.1). In the TOOTH study, of 542 participants, 22% were frail and 32% had mobility limitations. Just over a tenth (13%) of respondents reported severe difficulty eating foods because of dental problems (not being able to eat ≥4 listed foods), and 30% had no remaining natural teeth.

**Table 1:** Participant characteristics and prevalence of oral health problems in the baseline sample of the Newcastle 85+ Study and the TOOTH Study

	85+ Study (*n* = 853)	Tooth Study (*n* = 542)
Age (years), mean (±SD)	85 (0.6)	87 (2.2)
Sex, n (%)		
Women	530 (62)	306 (56)
Men	323 (38)	236 (44)
Education (years), mean (±SD)	14.6 (±1.1)	11 (±3.2)
Social class, n (%)		N/A
Higher managerial	191 (23)	
Intermediate occupations	111 (13)	
Routine and manual occupations	543 (64)	
Smoking, n (%)		
Never smoked and occasional smoker	330 (39)	316 (61)
Current smoker	45 (5)	36 (7)
Ex-regular smoker	473 (56)	170 (32)
BMI, mean (±SD)	24.5 (±4.4)	21.4 (±3.2)
SMMSE, mean (±SD)	25.8 (±5.5)	25.9 (±4.3)
History of cardiovascular disease, n (%)	409 (48)	121 (22)
History of hypertension, n (%)	483 (57)	443 (82)
History of diabetes, n (%)	112 (13)	86 (16)
Frailty		
Frail, n (%)	226 (28)	120 (22)
Pre-frail, n (%)	433 (54)	339 (63)
Robust, n (%)	144 (18)	83 (15)
Mobility limitations, n (%)	483 (57)	170 (32)
Grip strength (kg), mean (±SD)	19.2 (±8)	19.5 (±5.8)
Gait speed (seconds), mean (±SD)	18.7 (±14.8)	15.2 (±8.5)
**Oral health measures**		
Difficulty eating		
Severe	77 (9)^a^	68 (13)^b^
Any trouble eating hard foods	297 (37)^c^	272 (51)^d^
Difficulty swallowing, n (%)	131 (16)	69 (13)
Dry mouth	462 (57)^e^	1.4 (1.1)^f^
Complete tooth loss, n (%)	450 (55)	160 (30)
At least 21 teeth, n (%)	71 (9)	92 (17)

*SD* Standard deviation, *SMMSE* Standardised mini mental state examination test. ^a^ Severe defined as could not eat at all ≥3 foods. ^b^ Severe defined as could not eat at all ≥4 foods. ^c^ Any trouble eating ≥3 hard foods. ^d^ Any trouble eating ≥2 hard foods. ^e^ Number of responses to any dry mouth symptom (%). ^f^ Mean (SD) of saliva volume (gm)

Tables 2 and 3 present results for the Newcastle 85+ Study. In age and sex-adjusted models (age-adjusted for brevity), difficulty swallowing and difficulty eating ≥3 hard foods were associated with higher odds of frailty, which remained on full adjustment (Table 2). All oral health markers were significantly associated with greater odds of having mobility limitations. These associations persisted, except for complete tooth loss, in models fully adjusted for socio-demographic characteristics, BMI, alcohol intake, smoking, and co-morbidities. Dry mouth symptoms, difficulty swallowing, both measures of difficulty eating, and complete tooth loss were also associated with slow gait speed in the fully-adjusted models (Table 3). Both measures of difficulty eating were additionally associated with increased odds of having weak grip strength.

**Table 2:** Odds ratios and 95% CI for the association of oral health markers with frailty and mobility limitations in older people in the Newcastle 85+ Study

Oral health markers	Frailty^**a**^			Mobility limitations^**b**^		
		Age-adjusted^c^	Fully-adjusted^d^		Age-adjusted^c^	Fully-adjusted^d^
	N (%)	OR (95% CI)	OR (95% CI)	N (%)	OR (95% CI)	OR (95% CI)
Dry mouth symptoms						
No	73 (21)	1.00	1.00	153 (44)	1.00	1.00
Yes	145 (32)	**1.70 (1.23,2.36)**	1.4 (0.92,2.11) ^e^	295 (64)	**2.26 (1.69,3.01)**	**1.76 (1.26,2.46)** ^e^
Difficulty swallowing						
No	165 (25)	1.00	1.00	347 (51)	1.00	1.00
Yes	55 (43)	**2.32 (1.56,3.45)**	**1.75 (1.09,2.80)**	102 (78)	**3.38 (2.17,5.26)**	**2.52 (1.56,4.08)**
Difficulty Eating						
Severe difficulty eating						
0 to 2 foods	189 (26)	1.00	1.00	390 (53)	1.00	1.00
3 to 10 foods	31 (41)	**1.83 (1.12,3.00)**	1.43 (0.78,2.61)	59 (78)	**2.87 (1.63,5.03)**	**2.89 (1.52,5.50)**
Any trouble eating hard foods						
0 to 2 foods	124 (23)	1.00	1.00	275 (50)	1.00	1.00
3 to 10 foods	96 (38)	**1.98 (1.43,2.76)**	**1.84 (1.26,2.70)**	174 (68)	**2.01 (1.46,2.75)**	**1.92 (1.35,2.73)**
Number of teeth						
≥ 21 teeth	13 (18)	1.00	1.00	24 (34)	1.00	1.00
1–20 teeth	26 (28)	1.41 (0.72,2.73)	1.39 (0.64,3.01)	61 (64)	**2.27 (1.31,3.92)**	**2.43 (1.31,4.50)**
0 teeth	20 (19)	**2.02 (1.07,3.83)**	1.84 (0.88,3.89)	49 (46)	**2.88 (1.69,4.90)**	**2.79 (1.49,5.00)**
Partial tooth loss						
≥ 21 teeth	13 (18)	1.00	1.00	24 (34)	1.00	1.00
< 21 teeth	208 (29)	1.76 (0.94,3.30)	1.65 (0.80,3.41)	429 (58)	**2.61 (1.56,4.38)**	**2.59 (1.44,4.65)**
Complete tooth loss						
1 or more teeth	139 (32)	1.00	1.00	270 (61)	1.00	1.00
No teeth	82 (22)	**1.53 (1.11,2.12)**	1.41 (0.95,2.07)	183 (49)	**1.48 (1.12,1.97)**	1.32 (0.95,1.83)

^a^Frailty status (Frail vs non-frail). ^b^Based on reported difficulty getting around in the house, or going up and down stairs/steps, or walking at least 400 yards. ^c^Adjusted for age and sex. ^d^Adjusted for age, sex, BMI, alcohol intake, smoking status, social class, cardiovascular disease, diabetes, hypertension, neuropsychiatric disease and other health conditions (cerebrovascular disease, respiratory disease, musculoskeletal disease, and cancer diagnosis in the last 5 years). ^e^Additionally adjusted for depression and taking medications with dry mouth as side effect

**Table 3:** Odds ratios and 95% CI for the associations of oral health markers with weak grip strength and slow gait speed in older people in the Newcastle 85+ Study

Oral health markers	Weak Grip Strength^**a**^			Slow Gait Speed^**b**^		
		Age-adjusted^c^	Fully-adjusted^d^		Age-adjusted^c^	Fully-adjusted^d^
	N (%)	OR (95% CI)	OR (95% CI)	N (%)	OR (95% CI)	OR (95% CI)
Dry mouth symptoms						
No	106 (31)	1.00	1.00	95 (28)	1.00	1.00
Yes	160 (35)	1.21 (0.89,1.63)	1.04 (0.74,1.48) ^e^	190 (43)	**1.91 (1.41,2.58)**	**1.64 (1.13,2.38)** ^e^
Difficulty swallowing						
No	210 (31)	1.00	1.00	217 (33)	1.00	1.00
Yes	58 (45)	**1.78 (1.22,2.61)**	1.36 (0.87,2.13)	70 (58)	**3.38 (2.17,5.26)**	**2.52 (1.56,4.08)**
Difficulty Eating						
Severe difficulty eating						
0 to 2 foods	229 (31)	1.00	1.00	247 (35)	1.00	1.00
3 to 10 foods	39 (51)	**2.30 (1.43,3.72)**	**1.89 (1.08,3.30)**	40 (55)	**2.28 (1.40,3.72)**	**2.39 (1.33,4.29)**
Any trouble eating hard foods						
0 to 2 foods	162 (29)	1.00	1.00	166 (31)	1.00	1.00
3 to 10 foods	106 (42)	**1.75 (1.27,2.39)**	**1.66 (1.16,2.36)**	121 (50)	**2.24 (1.64,3.07)**	**2.44 (1.70,3.51)**
Number of teeth						
≥ 21 teeth	17 (24)	1.00	1.00	18 (25)	1.00	1.00
1–20 teeth	27 (29)	1.43 (0.79,2.62)	1.39 (0.72,2.65)	35 (38)	1.39 (0.77,2.50)	1.30 (0.67,2.55)
0 teeth	37 (35)	**1.84 (1.03,3.30)**	1.85 (0.97,3.51)	27 (25)	**2.08 (1.18,3.70)**	1.86 (0.97,3.60)
Partial tooth loss						
≥ 21 teeth	253 (35)	1.00	1.00	271 (38)	1.00	1.00
< 21 teeth	17 (24)	1.67 (0.95,2.94)	1.62 (0.87,3.00)	18 (25)	**1.77 (1.02,3.09)**	1.59 (0.84,3.01)
Complete tooth loss						
1 or more teeth	160 (37)	1.00	1.00	176 (42)	1.00	1.00
No teeth	110 (30)	**1.37 (1.01,1.85)**	1.42 (1.00,2.02)	113 (31)	**1.60 (1.19,2.16)**	**1.50 (1.05,2.14)**

^a^ Defined as values falling in the lowest tertile of the grip strength distribution. ^b^Defined as values falling in the highest tertile of the TUG distribution. ^c^ Adjusted for age and sex. ^d^Adjusted for age, sex, BMI, alcohol intake, smoking status, social class, cardiovascular disease, diabetes, hypertension, neuropsychiatric disease and other health conditions (cerebrovascular disease, respiratory disease, musculoskeletal disease, and cancer diagnosis in the last 5 years). ^e^Additionally adjusted for depression and taking medications with dry mouth as side effect

Tables 4 and 5 present associations for the TOOTH study. In this population, both measures of difficulty eating were associated with frailty and mobility limitations even after adjustment for confounders. Odds ratios for those who had severe difficulty eating were 1.84 (95%CI 1.01–1.36) for frailty and 2.42 (95%CI 1.17–5.03) for mobility limitations. Difficulty eating ≥2 or more hard foods was also associated with greater risk of slow gait speed (odds ratio = 1.98; 95%CI 1.31–3.01). Dry mouth was not significantly associated with frailty or physical function measures. The odds of frailty were increased by about two-thirds for participants with no natural teeth compared to those having some teeth (OR = 1.67, 95% CI 1.05–2.67).

**Table 4:** Odds ratios and 95% CI for the associations of oral health markers with frailty and mobility limitations in older people in the TOOTH Study

Oral health markers	Frailty^**a**^			Mobility limitations^**b**^		
		Age-adjusted^c^	Fully-adjusted^d^		Age-adjusted^c^	Fully-adjusted^d^
	N (%)	OR (95% CI)	OR (95% CI)	N (%)	OR (95% CI)	OR (95% CI)
Dry mouth						
No	51 (18)	1.00	1.00	23 (8)	1.00	1.00
Yes	30 (21)	1.26 (0.75, 2.11)	1.32 (0.74, 2.35) ^e^	15 (10)	1.35 (0.65, 2.79)	1.49 (0.68, 3.27) ^e^
Difficulty swallowing						
No	96 (21)	1.00	1.00	56 (12)	1.00	1.00
Yes	19 (28)	1.55 (0.88, 2.74)	1.51 (0.81, 2.82)	17 (25)	**2.31 (1.20, 4.45)**	2.07 (0.96, 4.42)
Difficulty Eating						
Severe difficulty eating						
0 to 3 foods	46 (16)	1.00	1.00	54 (12)	1.00	1.00
4 to 15 foods	67 (29)	**1.89 (1.08, 3.33)**	**1.84 (1.01, 3.36)**	17 (25)	**1.99 (1.02, 3.90)**	**2.42 (1.17, 5.03)**
Any trouble eating hard foods						
0 to 1 foods	40 (16)	1.00	1.00	19 (7)	1.00	1.00
2 to 5 foods	75 (28)	**1.95 (1.27, 3.00)**	**1.94 (1.23, 3.05)**	54 (20)	**2.69 (1.52, 4.75)**	**2.84 (1.53, 5.26)**
Number of teeth						
≥ 21 teeth	17 (18)	1.00	1.00	9 (10)	1.00	1.00
1–20 teeth	52 (19)	1.02 (0.55, 1.88)	0.94 (0.48, 1.83)	33 (12)	1.13 (0.51, 2.50)	1.34 (0.51, 3.54)
0 teeth	46 (29)	1.53 (0.81, 2.88)	1.77 (0.87, 3.61)	31 (19)	1.72 (0.75, 3.92)	2.27 (0.82, 6.30)
Partial tooth loss						
≥ 21 teeth	17 (18)	1.00	1.00	9 (10)	1.00	1.00
< 21 teeth	98 (22)	1.25 (0.70, 2.23)	0.94 (0.65, 2.27)	64 (15)	1.33 (0.62, 2.83)	1.60 (0.64, 3.99)
Complete tooth loss						
1 or more teeth	69 (19)	1.00	1.00	42 (11)	1.00	1.00
No teeth	46 (29)	**1.63 (1.06, 2.55)**	**1.67 (1.05, 2.67)**	31 (19)	1.49 (0.87, 2.54)	1.54 (0.85, 2.78)

^a^Frailty status (Frail vs non-frail). ^b^Based on reported reported difficulty getting around in the house, or going up and down stairs/steps, or walking at least 400 yards. ^c^Adjusted for age and sex. ^d^Adjusted for age, sex, BMI, alcohol intake, smoking status, education level, cardiovascular disease, diabetes, and hypertension. ^e^Additionally adjusted for depression

**Table 5:** Odds ratios and 95% CI for the associations of oral health markers with weak grip strength and slow gait speed in older people in the TOOTH Study

Oral health markers	Weak Grip Strength^**a**^			Slow Gait Speed^**b**^		
		Age-adjusted^c^	Fully-adjusted^d^		Age-adjusted^c^	Fully-adjusted^d^
	N (%)	OR (95% CI)	OR (95% CI)	N (%)	OR (95% CI)	OR (95% CI)
Dry mouth						
No	93 (32)	1.00	1.00	100 (35)	1.00	1.00
Yes	49 (34)	1.14 (0.74, 1.76)	1.12 (0.70, 1.80) ^e^	48 (34)	0.97 (0.63, 1.49)	0.95 (0.60, 1.52) ^e^
Difficulty swallowing						
No	158 (34)	1.00	1.00	132 (33)	1.00	1.00
Yes	23 (34)	0.95 (0.55, 1.67)	0.88 (0.48, 1.60)	23 (45)	1.67 (0.91, 3.05)	1.82 (0.97, 3.43)
Difficulty Eating						
Severe difficulty eating						
0 to 3 foods	147 (32)	1.00	1.00	129 (32)	1.00	1.00
4 to 15 foods	33 (49)	**1.79 (1.04, 3.06)**	1.74 (0.99, 3.07)	26 (47)	1.64 (0.91, 2.95)	1.71 (0.93, 3.14)
Any trouble eating hard foods						
0 to 1 foods	78 (30)	1.00	1.00	61 (27)	1.00	1.00
2 to 5 foods	103 (38)	1.29 (0.89, 1.97)	1.26 (0.85, 1.86)	94 (42)	**1.91 (1.28, 2.85)**	**1.98 (1.31, 3.01)**
Number of teeth						
≥ 21 teeth	27 (29)	1.00	1.00	28 (36)	1.00	1.00
1–20 teeth	93 (34)	1.21 (0.72, 2.04)	1.30 (0.74, 2.30)	74 (31)	1.13 (0.63, 2.04)	1.13 (0.61, 2.11)
0 teeth	59 (37)	1.22 (0.69, 2.16)	1.24 (0.66, 2.33)	53 (40)	0.79 (0.46, 1.36)	0.72 (0.41, 1.27)
Partial tooth loss						
≥ 21 teeth	27 (29)	1.00	1.00	28 (36)	1.00	1.00
< 21 teeth	152 (35)	1.22 (0.74, 2.01)	1.27 (0.74, 2.30)	127 (34)	0.89 (0.53, 1.49)	0.83 (0.49, 1.40)
Complete tooth loss						
1 or more teeth	120 (33)	1.00	1.00	102 (32)	1.00	1.00
No teeth	59 (37)	1.07 (0.71, 1.60)	1.02 (0.66, 1.57)	53 (40)	1.32 (0.86, 2.02)	1.35 (0.86, 2.12)

^a^ Defined as values falling in the lowest tertile of the grip strength distribution. ^b^Defined as values falling in the highest tertile of the TUG distribution. ^c^ Adjusted for age and sex. ^d^Adjusted for age, sex, BMI, alcohol intake, smoking status, education level, cardiovascular disease, diabetes, and hypertension. ^e^Additionally adjusted for depression

## Discussion

We have used two studies of the oldest old (aged > 85 years) in England and Japan to examine the association between subjective and objective oral health markers with well-established indicators of physical function, frailty and disability in very old ages. The findings add further evidence for the interlinkages of oral health with poor physical functioning and frailty in older people [6]. Self-reported oral health measures such as level of difficulty eating specific foods because of dental problems were consistently related to physical function and frailty measures; tooth loss was also associated with mobility limitations and slow gait speed.

Complete tooth loss and number of teeth is thought to influence functional disability and frailty in the very old through its potential role in nutritional and inflammatory pathways impacting on muscle loss and decline in physical function [8]. Our results showed that number of teeth or lack of teeth was associated with mobility limitations and slow gait speed in the Newcastle 85+ Study. Having no natural teeth compared with having any number of teeth was associated with greater odds of frailty in the Japanese study. Some earlier studies in older people have not found significant associations of number of teeth with mobility limitations, gait speed and grip strength [18, 30, 31]. Although the number of teeth present may influence muscle strength through impacting on nutritional intake, e.g. protein intake, muscle strength will also be affected by levels of physical activity and sedentary lifestyles [32], making it harder to disentangle the potential effects of oral health.

We observed significant associations between complete tooth loss and frailty score in the Japanese TOOTH study population, but found no significant associations in the Newcastle 85+ Study. Two previous studies have also found higher odds of frailty in edentulous subjects in Brazilian and British samples [12, 14]. The lack of significant associations observed in the Newcastle 85+ Study could be due to the relatively high prevalence of edentulism in this population, compared with the TOOTH study. It is likely there are differences in the causes underlying tooth loss in the English and Japanese study populations. In the Newcastle 85+ Study a number of participants had lost teeth at a young age possibly due to treatment practices prevalent at the time. Participants with long-term tooth loss may have adapted to their condition, reducing the impact on the quality of their diets [33], and in turn, its implications for frailty. In addition, tooth loss in this sample may not reflect periodontal or other inflammatory processes that are related to developing frailty. Measures of periodontal disease were not collected in these studies, hindering the exploration of its potential mediating role in the association of tooth loss with frailty.

Dry mouth in older people is commonly due to medications taken for comorbidities (diabetes, hypertension and depression). Dry mouth symptoms were significantly independently associated with slow gait speed and mobility limitations in the Newcastle 85+ Study. Previous studies have also reported independent associations of dry mouth symptoms with frailty, mobility and muscle strength [14, 16]. In the TOOTH study, dry mouth was not associated with frailty or physical function measures. Dry mouth in this study was based on a proxy measure using salivary volume. It is possible that self-reported measures of dry mouth are better indicators of its impact on difficulty eating and nutritional status.

Our findings additionally showed that self-reported difficulty eating due to dental problems and difficulty swallowing, were associated with frailty, mobility limitations, gait speed and grip strength. Similar associations have been reported between difficulty eating and mobility problems [14–16].

A strength of our study is that it is based on largely representative samples of community-dwelling oldest old populations in England and Japan. The studies have detailed well-characterised information on oral health, physical function and covariates, which enabled the present associations to be investigated. Limitations include the cross-sectional nature of the investigations, which precluded the possibility of exploring prospective associations of oral health with decline in frailty and physical function. Therefore, results presented are not evidence of causal associations. Nonetheless, oral health and physical functioning in older people are likely to have common underlying origins in sociodemographic, biologic and lifestyle risk factors that would make disentangling of these effects difficult even with longitudinal samples [6]. Moreover, the study samples were from one city (Newcastle in England, and Tokyo in Japan), which may limit the generalisability of findings to other populations.

## Conclusion

We found that in addition to tooth loss, measures of perceived difficulty eating were associated with markers of poor physical function in two different population samples of the oldest old. With an increasing proportion of ageing populations globally, there is a greater recognition of the public health challenges of healthy ageing and frailty in later life. Our findings suggest that oral health problems in older people pose additional challenges and need to be recognised as part of achieving healthy ageing and maintaining good physical function. Markers of oral health could be of use to researchers and practitioners looking to understand and alleviate the burden of frailty and limitations in physical function of ageing populations. Further research from prospective studies is needed to understand the associations longitudinally between oral health problems and frailty in later life. Pathways underlying oral health and the development of poor physical function in older adults also merit further research.

## Supplementary Information

**Additional file 1.**

## Data Availability

The data that support the findings of this study are available from Tokyo Oldest Old Survey on Total Health study (http://www.keio-centenarian.com/english/research/tooth) and the Newcastle 85+ study (https://research.ncl.ac.uk/85plus/) but restrictions apply to the availability of these data, which were used under license for the current study, and so are not publicly available. Data are however available from the authors upon reasonable request and with permission from the above data owners.

## Abbreviations

TOOTH: Tokyo Oldest Old Survey on Total Health
FFS: Fried Frailty Phenotype Score
ADL: Activities of daily living
GPRR: General practice record reviews
TUG test: Timed-up-and-go test
CVD: Cardiovascular disease
TIA: Transient ischaemic attack
GDS-15: Geriatric Depression Scale
BMI: Body mass index
OR: Odds ratio
