# Supplementary material for: Associations of poor oral health with frailty and physical functioning in the oldest old: results from two studies in England and Japan
Source: BMC Geriatr. 2021 Mar 18;21:187. doi: 10.1186/s12877-021-02081-5 (PMC7977173; doi:10.1186/s12877-021-02081-5)
Supplement: Supplementary file 1 — Additional file 1. [file 12877_2021_2081_MOESM1_ESM.docx]

**Associations of poor oral health with frailty and physical functioning in the oldest old: results from two studies in England and Japan**

**Supplementary methods**

# Frailty status

Table A.1 presents the items of the Fried Frailty status (FFS) measures used in the two study populations of the Newcastle 85+ Study and the TOOTH Study. The FFS indicator was constructed from an adaptation of the measure derived from the Cardiovascular Health Study prospective observational cohort of men and women over 65 years of age [1]. “Frail” defined as meeting at least three of the five criteria detailed below. FFS was set to missing when three or more of the items comprising the score were missing.

Table A 1. Items comprising the frailty score used in the study

|  | **Newcastle 85+ study** |  |  | **TOOTH study** |  |
| --- | --- | --- | --- | --- | --- |
| **Criterion (yes/no)** | **Response item/functional measure** | **Frailty threshold** | **Notes** | **Response item/functional measure** | **Frailty threshold** |
| Shrinking | BMI (kg/m^2^) | BMI <18.5 kg/m^2^ (underweight). | Participants with no weight data because of "participant unable to stand" or "frailty/fatigue" coded as “frail”. | Answer to the item “have you lost 3kg in the past year?” | “Yes” to weight loss question. |
| Poor endurance/  Energy | Answers to the items "do you feel full of energy" (response options “yes” or “no”) and “'During the last 4 weeks how often rested in bed during day?” (response options: “every day”, “every week”, “once”, “not at all”). Geriatric depression scale (15 item) [2] | - "do you feel full of energy": “No”   And   - Frequency of resting during the day: “every day” or “every week” | Coded as missing if any of the answers to the two questions was missing. | Answer to the item “I have felt active and vigorous” [3] | Score of 0-2 defined as frailty. |
| Low physical activity | Reported frequency of moderately energetic and very energetic physical activity (response options: “≥3 times per week”, “1-2 times per week”, “1-3 times per month”, “hardly ever/never”. | - Very energetic physical activity: “hardly ever/ never”   And   - Moderately energetic physical activity: “hardly ever/never” | Missing values reflect missing responses to both questions. | Walk and exercise time in a week by self-reported in questionnaires. | Lowest 20% of time stratified by sex defined as frail. |
| Weakness | Hand grip strength (kg) measure from hand held dynamometer (TKK 5401, Takei, Japan). Two measurements per hand. Average of 2 highest values across 4 values. | Calculated from lowest 20%; stratified by gender and body mass index (BMI kg/m^2^) quartiles [1]:  Men  BMI≤24, frailty cut point ≤29  BMI 24.1-26, frailty cut point ≤30  BMI 26.1-28, frailty cut point ≤30  BMI>28, frailty cut point ≤32  Women  BMI≤23, frailty cut point ≤17  BMI 23.1-26, frailty cut point ≤17.3  BMI 26.1-29, frailty cut point ≤18 | Participants with missing information on grip strength because of “fraitly/fatigue” coded as “frail”.  Only cases with missing values in all 4 grip-strength measurements set to missing. | Hand grip strength (kg) measure from hand held dynamometer (Tanita 6103, Tanita cooperation, Tokyo, Japan). | As per Newcastle 85+ method |
| Slow walking speed | Time taken (in seconds) to complete “time up and go” (TUG) test. | TUG ≥19 seconds | Missing responses because of participant “severely limited mobility” or “mobility limitations” coded as “frail”. | Time taken (in seconds) to complete “time up and go” (TUG) test. For subjects who could not perform the TUG-test, coded as frail using a question about 50 m walkability: can walk for oneself = 1, can walk with assistance (e.g., walking frame) = 2, can move 50 m with a wheelchair = 3, can’t move 50 m = 4. | The lower 20% for each gender in TUG test are frailty.  Responding 2, 3 or 4 to walkability question. |

# References

[1] Fried, L. P., Tangen, C. M., Walston, J., Newman, A. B.*, et al.*, Frailty in Older Adults: Evidence for a Phenotype. *The Journals of Gerontology: Series A* 2001, *56*, M146-M157.

[2] Yesavage, J. A., Sheikh, J. I., 9/Geriatric Depression Scale (GDS). *Clinical Gerontologist* 1986, *5*, 165-173.

[3] Bradley, C., Lewis, K. S., Measures of Psychological Well-being and Treatment Satisfaction Developed from the Responses of People with Tablet-treated Diabetes. *Diabetic Medicine* 1990, *7*, 445-451.
